# Supplementary material for: Establishment and maintenance of motor neuron identity via temporal modularity in terminal selector function
Source: eLife. 2020 Oct 1;9:e59464. doi: 10.7554/eLife.59464 (PMC7529460; doi:10.7554/eLife.59464)
Supplement: Supplementary file 1. [file elife-59464-supp1.docx]

**Supplementary file 1: Analysis of UNC-3 targets for direct UNC-3 binding.**

|  | **Terminal**  **identity**  **gene** | **Previously known direct target** | **ChIP-Seq**  **peaks** | **Intron** | **ATG upstream** (<1kb) | **ATG**  **upstream**  (1-2kb) | **ATG upstream** (2-3kb) | **ATG upstream** (>3kb) |
| --- | --- | --- | --- | --- | --- | --- | --- | --- |
| *1* | *unc-17* | Yes | Yes | + | - | - | + | + |
| *2* | *cho-1* | Yes | Yes | + | - | - | - | + |
| *3* | *ace-2* | Yes | Yes | - | - | - | - | + |
| *4* | *acr-2* | Yes | Yes | - | + | - | - | - |
| *5* | *acr-14* | Yes | Yes | - | + | - | - | - |
| *6* | *del-1* | Yes | Yes | - | + | - | + | + |
| *7* | *glr-4* | Yes | Yes | - | + | + | - | + |
| *8* | *madd-4* | Yes | Yes | + | - | - | + | - |
| *9* | *unc-129* | Yes | Yes | - | + | - | - | - |
| *10* | *acr-16* | Yes | No | - | - | - | - | - |
| ***Summary*** | | **9/10** previously known direct targets show UNC-3 binding based on ChIP-Seq | | | | | | |
| *11* | *acr-5* | No | Yes | - | + | - | - | - |
| *12* | *acr-15* | No | Yes | - | + | - | - | - |
| *13* | *unc-63* | No | Yes | - | - | + | - | - |
| *14* | *gar-2* | No | Yes | + | - | + | - | - |
| *15* | *trp-1* | No | Yes | - | - | + | - | - |
| *16* | *unc-77* | No | Yes | + | - | + | - | - |
| *17* | *unc-8* | No | Yes | + | - | - | - | - |
| *18* | *inx-12* | No | Yes | - | - | - | - | + |
| *19* | *acc-4* | No | Yes | - | + | + | - | - |
| *20* | *gbb-1* | No | Yes | - | + | - | - | + |
| *21* | *kvs-1* | No | Yes | + | - | - | - | + |
| *22* | *dbl-1* | No | Yes | - | + | + | - | + |
| *23* | *tig-2* | No | Yes | - | + | + | - | - |
| *24* | *nlp-21* | No | Yes | + | - | + | - | - |
| *25* | *unc-40* | No | Yes | + | + | - | - | - |
| *26* | *max-1* | No | Yes | + | + | + | - | - |
| *27* | *F39B2.8* | No | Yes | + | + | - | - | - |
| *28* | *dop-1* | No | Yes | - | - | - | - | + |
| *29* | *tsp-7* | No | Yes | - | + | - | - | - |
| *30* | *rig-4* | No | Yes | - | - | - | + | - |
| *31* | *F29G6.2* | No | Yes | - | + | - | - | - |
| *32* | *F55C12.4* | No | Yes | - | + | - | + | + |
| *33* | *tmc-1* | No | Yes | - | - | + | + | - |
| *34* | *twk-7* | No | Yes | + | + | + | + | - |
| *35* | *twk-13* | No | Yes | + | - | - | - | + |
| *36* | *twk-40* | No | Yes | + | + | - | - | - |
| *37* | *twk-43* | No | Yes | + | - | - | - | - |
| *38* | *acr-21* | No | Yes | - | + | - | - | - |
| *39* | *glr-5* | No | Yes | - | + | + | - | - |
| *40* | *exp-1* | No | Yes | + | + | - | - | - |
| *41* | *mig-13* | No | Yes | + | + | - | + | + |
| *42* | *ace-4* | No | Yes | + | - | - | - | - |
| *43* | *ddr-2* | No | Yes | - | + | - | - | - |
| *44* | *unc-53* | No | Yes | + | + | + | - | - |
| *45* | *flp-18* | No | Yes | + | + | - | - | - |
| *46* | *lgc-36* | No | Yes | - | + | - | - | - |
| *47* | *slo-2* | No | Yes | + | + | - | - | - |
| *48* | *nrx-1* | No | Yes | + | - | - | - | + |
| *49* | *lgc-55* | No | No | - | - | - | - | - |
| *50* | *itr-1* | No | No | - | - | - | - | - |
| *51* | *avr-15* | No | No | - | - | - | - | - |
| *52* | *ace-3* | No | No | - | - | - | - | - |
| *53* | *cog-1* | No | No | - | - | - | - | - |
| ***Summary*** | | **38/43** previously known *unc-3*-dependent genes show UNC-3 binding based on ChIP-Seq | | | | | | |
